# Seroprevalence of Hepatitis E virus infection in the Irish pig population

**DOI:** 10.1186/s13620-015-0036-3

**Published:** 2015-05-08

**Authors:** Michael O’Connor, Sarah-Jayne Roche, Dónal Sammin

**Affiliations:** Department of Agriculture, Food and the Marine Laboratories, Backweston, Celbridge, Co., Kildare, Ireland

**Keywords:** Hepatitis, Hepatitis E, Hepatitis E Virus, Pigs, Zoonosis, Epidemiology

## Abstract

**Background:**

Hepatitis E is an acute viral disease of humans, occurring in explosive outbreaks in the developing world and as sporadic cases in returning travellers. Increasing recognition of indigenous transmission in Western countries suggests a zoonotic source of infection, most likely pigs. To determine if hepatitis E virus is present in Irish pigs, sera from 330 animals were examined for antibodies using a commercially available ELISA.

**Findings:**

Antibodies were detected in 89 pigs (27%) in 13 herds (81%).

**Conclusions:**

Hepatitis E virus is present in most Irish pig herds and in many animals within these herds.

## Findings

Hepatitis E is now considered to be the most common type of acute viral hepatitis occurring in people across the globe [1]. Hepatitis E virus (HEV), the causative agent, is a member of the *Hepevirus* genus in the *Hepeviridae* family. It contains a single-stranded RNA genome within an icosahedral capsid 32–34 nm in diameter. Four distinct genotypes (HEV1-4) are currently recognised in affected persons. HEV1 and 2 appear to be restricted to humans whereas HEV3 and 4 can also infect other animal species, including pigs. HEV infection (genotypes 1 and 2) is endemic in many developing countries and large outbreaks of disease have been associated with poor sanitation and faecal contamination of water. Highest mortality occurs in pregnant women and people with underlying chronic liver disease. Hepatitis E also occurs sporadically in the developed world, especially in middle-aged or elderly men. Travel-associated HEV1/2 infections are occasionally found in persons returning from endemic regions but increasing numbers of indigenous cases attributable to HEV3 infection have been diagnosed in recent years. The latter has suggested the possibility of zoonotic transmission from pigs or other animal reservoirs [1,2], whilst other transmission routes such as via transfusion of blood products from viraemic persons have also been demonstrated [3]. One particular concern is that HEV3 can cause persistent chronic infection and poor clinical outcomes in immuno-compromised individuals [1]. Increasing interest in this “One Health” issue has prompted us to set down the findings of a limited survey undertaken in 2011 to quantify the extent of exposure of the Irish pig population to HEV infection.

A subset of sera from breeding pigs (gilts, sows and boars) was assembled from submissions received for Aujeszky’s Disease Surveillance by the Department of Agriculture, Food and the Marine Laboratories in 2010/2011. This comprised sera from at least five and in most cases 15 pigs (range 5-60 pigs) from each of 16 herds located across nine Irish counties. In total, sera from 330 pigs were tested for HEV antibody using the commercially available *PrioCHECK HEV Ab porcine ELISA,* which has a sensitivity and specificity of greater than 90%. According to the manufacturer’s instructions, sera with an OD 1.2 times that of the cut-off control were regarded as positive, while those with an OD between both values were regarded as doubtful. Eighty-nine pigs (27%) in 13 herds (81%) were seropositive, with ODs between 1.63 and 4.27 that of the cut-off control. Only one of the 14 sera classified as doubtful originated in an otherwise antibody-negative herd. In those herds in which one or more seropositive pigs were identified, between 7 and 70% of the animals that were tested had antibodies to HEV, denoting exposure to the virus at some stage during their lifetime.

These findings were not surprising given that HEV infection appears to be universal in pigs [2], with a high prevalence of exposure to the virus in intensively managed herds. To cite just two other studies from elsewhere – 92% of Danish sow herds and 73% of animals therein were found to be seropositive [4] whilst a preliminary report of a more recent UK study indicated that 93% of 640 pigs sampled at slaughter were seropositive [5]. Infection with HEV3 (and in some regions HEV4) occurs naturally in pigs but they appear to be resistant to infection by HEV1 and HEV2; the virus has been shown to cause microscopic hepatic and enteric lesions in experimentally-infected pigs but infection is invariably subclinical [2]. Infection most usually occurs during the post-weaning phase of the production cycle resulting in a higher seroprevalence in animals that are more than four months old, but wide variations in within-herd seroprevalence would appear to be the norm in HEV-infected herds [6].

Several studies [6,7] have shown an association between the occurrence of hepatitis E and consumption of raw or undercooked animal-derived foods which are presumed to have been the source of virus, but there are few documented cases [8,9] providing definitive evidence of such a link. In addition, there are a number of studies [10-12] which suggest that people who have direct contact with pigs, including veterinarians, are at higher risk of acquiring HEV infection than the general population. The background to the UK study cited above was a significant increase in the number of indigenous clinical cases of hepatitis E diagnosed in England and Wales over the last decade associated with the emergence of a different subtype (group 2) of HEV3 which now accounts for two-thirds of human cases [13]. Consequently the study on UK pigs also involved virological screening – almost 6% of the pigs that were sampled and tested in that study had detectable levels of HEV RNA in blood with 1% estimated to have a significant viraemia - i.e. where there was likely to be sufficient viable virus in blood and tissues to pose a risk of transmission. However, all of the viruses (viral RNA) identified in these pigs were genotyped as group 1 of HEV3 – i.e. it would appear that the majority of the indigenous cases of hepatitis E cannot be attributable to infection acquired from UK pigs. Therefore, further studies on other potential sources of human exposure to HEV would appear to be warranted. As HEV-infected pigs shed the virus in faeces and may harbour virus in tissues, any risk to human health might be mitigated by taking hygienic precautions such as careful hand-washing after contact with pigs and their by-products [2] and by thoroughly cooking pork products and offal [14].

## References

[1] Kamar N, Dalton HR, Abravanel F, Izopet J (2014). Hepatitis E virus infection. Clin Microbiol Rev.

[2] Meng XJ (2010). Hepatitis E virus: Animal reservoirs and zoonotic risk. Vet Microbiol.

[3] Hewitt PE, Ijaz S, Brailsford SR, Brett R, Dicks S, Haywood B (2014). Hepatitis E virus in blood components: a prevalence and transmission study in southeast England. Lancet.

[4] Breum SO, Hjulsager CK, de Deus N, Segalés J, Larsen LE (2010). Hepatitis E virus is highly prevalent in the Danish pig population. Vet Microbiol.

[5] Tedder RS. Hepatitis E virus infection in UK pigs at the time of slaughter. 2014. http://www.defra.gov.uk/ahvla-en/science/bact-food-safety/2013-pig-abattoir-study.

[6] Pavio N, Meng X-J, Renou C (2010). Zoonotic hepatitis E: animal reservoirs and emerging risks. Vet Res.

[7] Miyamura T. Hepatitis E virus infection in developed countries. Virus Res. 2011;161:40–6. 10.1016/j.virusres.2011.03.006 21443914

[8] Matsuda H, Okada K, Takahashi K, Mishiro S (2003). Severe hepatitis E virus infection after ingestion of uncooked liver from a wild boar. J Infect Dis.

[9] Tei S, Kitajima N, Takahashi K, Mishiro S (2003). Zoonotic transmission of hepatitis E virus from deer to human beings. Lancet.

[10] Drobeniuc J, Favorov MO, Shapiro CN, Bell BP, Mast EE, Dadu A (2001). Hepatitis E antibody prevalence among persons who work with swine. J Infect Dis.

[11] Meng XJ, Wiseman B, Elvinger F, Guenette DK, Toth TE, Engle RE (2002). Prevalence of antibodies to hepatitis E virus in veterinarians working with swine and in normal blood donors in the United States and other countries. J Clin Microbiol.

[12] Chaussade H, Rigaud E, Allix A, Carpentier A, Touzé A, Deizescaux D (2013). Hepatitis E virus seroprevalence and risk factors for individuals in working contact with animals. J Clin Virol.

[13] Ijaz S, Said B, Boxall E, Smit E, Morgan D, Tedder RS. Indigenous hepatitis E in England and Wales from 2003 to 2012: evidence of an emerging novel phenotype of viruses. J Infect Dis. 2014;209:1212–8. 10.1093/infdis/jit652 24273173

[14] Guidance provided by the Food Safety Authority of Ireland at https://www.fsai.ie/.

